# Strengthening the use of regulatory policy measures for prevention of NCDs in Europe through the JA PreventNCD project

**DOI:** 10.1177/14034948251374402

**Published:** 2025-09-20

**Authors:** Arnfinn Helleve, Maria João Gregório, Daniel Bergsvik, Anette Kocbach Bølling, Valentina De Cosmi, Neza Fras, Mojca Gabrijelčič Blenkuš, Karine Gallopel-Morvan, Line Småstuen Haug, Thomas Karlsson, Mikaela Lindeman, Betina Bergmann Madsen, Marco Silano, Taina Siponen, Sabrina Teyssier, Tord Finne Vedøy, Karine Vin, Johan Øvrevik

**Affiliations:** 1 Centre for Evaluation of Public Health Measures, Norwegian Institute of Public Health, Norway; 2 National Programme for the Promotion of Healthy Eating, Directorate-General of Health, Portugal; 3 Department of Alcohol, Tobacco and Drugs, Norwegian Institute of Public Health, Norway; 4 Air Quality and Noise, Norwegian Institute of Public Health, Norway; 5 Department of Food Safety, Italian National Institute of Health, Italy; 6 National Institute of Public Health, Slovenia; 7 EHESP, CNRS, Inserm, Arènes - UMR6051, RSMS - U1309, Rennes University, Rennes, France; 8 Department of Food Safety and Centre for Sustainable Diets, Norwegian Institute of Public Health, Norway; 9 Finnish Institute for Health and Welfare, Finland; 10 Copenhagen Municipality, Denmark; 11 Department of Cardiovascular, Endocrine–metabolic Diseases and Aging, Italian National Institute of Health, Italy; 12 National Research Institute for Agriculture, Food and Environment, National Centre for Scientific Research, University of Grenoble Alpes, France; 13 French Agency for Food, Environmental and Occupational Health & Safety (ANSES), France; 14 Division of Climate and Environmental Health, Norwegian Institute of Public Health, Norway

**Keywords:** JA PreventNCD, structural measures, fiscal policies, evidence-based NCD prevention

## Abstract

**Aims:**

The Joint Action project on Cancer and other Non-communicable Diseases (NCDs) prevention, Action on Health Determinants, includes a dedicated workstream on structural and population-level interventions. The overarching objective of this workstream is to strengthen the compliance, coherence, implementation and enforcement of evidence-based regulatory measures that support governmental efforts to reduce the burden of NCDs.

**Methods:**

The workstream adopts a multi-method approach, informed by existing academic literature and previous European studies. Key methodologies include policy mapping, evidence reviews, behavioural assessments, policy impact modelling, and pilot testing. Governmental alcohol and tobacco policies will be evaluated using comparative policy scales, while the health and economic impacts of health taxation policies will be projected through and microsimulation modelling. Nutrient profile modelling and food composition databases will be developed to inform strategies for food reformulation. The effectiveness of labelling interventions will be examined. Tools for monitoring digital marketing exposure will be developed, and the impact of environmental policy impact will be assessed.

**Expected results:**

The workstream is expected to deliver comprehensive policy analyses, demonstrate the potential impact of health taxation, propose harmonized nutrient profiling frameworks, assess the effectiveness of food and alcohol labelling practices and contribute to the development of cross-national structures for public food procurement. Additionally, it will provide guidance on the implementation of effective measures and evaluate divergences in national policy approaches across Europe.

**Conclusions:**

**The workstream will generate actionable evidence and documentation to inform and support public policy processes, thereby contributing to reductions in the burden of preventable disease across the region.**

## Background

A wide range of evidence-based policy interventions [1] are available for adoption and implementation by countries to address modifiable risk factors like tobacco use, alcohol use and unhealthy diets, and potentially reduce the burden of non-communicable diseases (NCDs) and cancers. Despite the universal relevance of NCD prevention in all countries, there is considerable cross-national variation in the extent and nature of policy implementation, whether in the policy domains of diet [2], physical activity, tobacco use [3] or alcohol consumption [4]. These policy disparities between countries highlight the potential for many European countries to strengthen, align and scale up their policy efforts. They also offer opportunities for shared learning, impact comparison and cross-country collaborations.

Policy variations across countries can be attributed to differences in political systems, national priorities, cultural contexts and the extent to which governments are influenced by lobbying activities [5]. Public policy making is a complex process involving a series of decisions, rather than a singular discrete choice; it also encompasses instances when decision making is avoided [6]. Although policy development is rarely linear, it can be conceptualised as comprising several stages: problem emergence, agenda setting, consideration of policy options, decision making, implementation and evaluation [6-8]. Policies to improve public health frequently intersect with other sectors, such as agriculture (e.g. food and wine production), trade (e.g. taxation) and housing (e.g. health equity). Environmental exposures, such as chemicals, noise and air pollution, also contribute to the prevalence of NCDs [9]. As a result, public discourse around these measures often extends beyond health effectiveness, encompassing ideological views, commercial interests and public opinion, all of which shape the policy-making environment.

Policy variation can also be explained by the nature of the policy interventions themselves. Many behavioural risk factors for NCDs are linked to high consumption of unhealthy, but legal, commodities like tobacco, alcohol, ultra-processed foods and beverages with high content of salt, sugar or fat. Some policies, such as providing recommendations and public information campaigns, are typically viewed as the least intrusive [10,11]. However, these measures rely on individual agency and may inadvertently exacerbate social inequalities [12]. While the provision of accurate and reliable information is essential, it must be complemented by structural measures that influence the availability, accessibility and affordability of healthier commodities and options. Regulatory and fiscal measures are therefore also required and recommended [1], though they are often seen as more intrusive and controversial.

In the European Union (EU) context, regulatory measures may be perceived as interfering with the free movement of goods, services, capital and people, and thus require careful assessments of their necessity and proportionality. Moreover, in liberal democracies, citizens and institutions are entitled to oppose policy measures that they believe to conflict with their own interests. At the same time, commercial actors employ a broad range of strategies to influence policy decision making in their favour [13]. There are numerous documented cases of food, alcohol and tobacco companies using lobbying tactics to obstruct or delay legal measures such as mandatory warnings labels, taxation and marketing regulations [14-16]. As a result, in many European countries, and many EU directives, regulatory measures are either inadequately implemented or not implemented at all.

The content of the workstream on regulatory policy measures within the EU-funded initiative *Joint Action on Cancer and other NCDs prevention – Action on Health Determinants* (JA PreventNCD) [17] aligns with the strategic objectives outlined in the *Healthier Together* guiding document [18] and *Europe’s Beating Cancer Plan* [19] and reflects shared interests and policy priorities among EU member states to advocate for the implementation of regulatory and fiscal measures to address health determinants, particularly those related to the consumption of alcohol, tobacco and unhealthy foods, as well as environmental exposures. Key policy instruments promoted include enforcement of EU tobacco control laws; improved compliance and enforcement of existing tobacco and alcohol regulations; food reformulation; public food procurement (PFP) standards; and restrictions on digital marketing of food, tobacco and alcohol. Additional priorities include strengthening the enforcement of bans of tobacco advertising, limiting cross-border purchases, increasing taxation on tobacco and related products, enhancing the effectiveness of alcohol warning labels, improving coherence of fiscal policies concerning alcohol and food products and promoting portion size reduction. Emphasis is placed on coherence, compliance, enforcement and implementation of existing policies, as well as generating new evidence to support and refine regulatory policy approaches to NCD prevention. These policy ambitions are consistent with those endorsed by the World Health Organization (WHO) [20].

This paper presents the objectives, methodological approaches and expected outcomes of the JA PreventNCD workstream dedicated to regulatory and fiscal policy measures, with the aim of supporting their use to reduce the burden of NCDs across Europe.

## Aims

The overall objective of the workstream is to strengthen the evidence base and enhance compliance, coherence, wider implementation and enforcement of fiscal and regulative policies across European countries to reduce the burden of NCDs, including cancers. The six specific objectives are to: (1) map and analyse policies and legislative frameworks related to trade of tobacco, alcohol, foods and non-alcoholic beverages, as well as exposure to environmental factors; (2) generate knowledge on the application of health taxes and fiscal policies aimed at enabling healthier behaviours; (3) promote food reformulation, sustainable PFP, food portion size standards and the use of a common nutrient profiling model to inform policy measures addressing unhealthy diet; (4) assess and monitor the impact of food and alcohol labelling for healthier behaviours; (5) support the implementation of policies that reduce the influence of harmful marketing; and (6) strengthen regulatory provisions related to pollution and exposure to hazardous substances. The equity dimensions of all the policy measures will be systematically assessed.

## Methods

The wide range of methodological approaches applied to achieve the six objectives of the workstream includes mapping and reviewing existing evidence, policies and behaviours: modelling policy impacts and pilot testing promising policy interventions.

For objective (1), we will map alcohol and tobacco/nicotine-related policies across European countries, compare the current policy landscape with the findings from earlier studies (e.g. Alice-RAP [21] alcohol and tobacco policy scales) and conduct expert consultations. The policy scale methodology will be further developed to better reflect the digital marketplace and digital regulatory solutions. Particular attention will be given to assessing the role of local autonomy and enforcement in alcohol and tobacco regulation. Regarding objective (2), the Centre for Health Economics & Policy Innovation at the Imperial College Business School has been contracted to review and evaluate current EU and national health taxation policies. They will model their potential impacts using a microsimulation model [22] applicable across European member states. We will collect and analyse evidence from selected countries on excise taxes to promote harmonized pricing of alcohol and tobacco products within the internal market and investigate the relationship between excise taxes, consumption prevalence and cross-border trade in neighbouring countries with substantial cross-border flows. We will also examine e-commerce, including domestic and cross-border online sales, and digital marketing of alcohol, tobacco and novel nicotine products in selected European countries.

For objective (3), we will conduct a survey of all EU member states to map and review existing nutrient profiling models that support nutrition-related policies at the EU level. The goal is to develop a harmonized nutrient profile model in collaboration with WHO Europe, to be tested in food databases in five countries. To support food reformulation policies, we will collect datasets from 12 selected countries, categorise them according to classifications established in the Best-ReMap project [23] and stored in a database hosted by EU’s Joint Research Centre [24]. Data on at least one of the five selected food groups will be updated during the Joint Action, depending on national capacities. These datasets will establish a baseline for the nutritional quality of the food supply in 19 countries and five food categories, as well as enable monitoring of food reformulation progress.

Additionally, we will carry out a situation analysis of existing legislation on PFP in European countries to support alignment with EU actions on sustainable food procurement. A survey in selected EU member states will assess the consistency between recommended food portion sizes by health authorities, those labelled by industry and those typically consumed. Based on these analyses, we aim to develop standard portion sizes by age group and product category to ensure the nutritional adequacy and prevent excessive energy intake or food waste.

A comprehensive mapping and analysis of national PFP legislation will also be conducted across EU member states, with a focus on promoting awareness and dissemination of sustainable public procurement practices, in alignment with European Commission actions. An EU Network of National Focal Points for PFP will be established, alongside regional and local-level networks. These networks will facilitate collaboration, knowledge sharing and professional development for public procurement professionals. In addition, we will develop guidance documents to support development of national strategies for creating tailored action plans and strategies for PFP, with a particular emphasis on the effective integration of sustainability as a criteria in the tender process.

For objective (4), we will first assess the effectiveness of new alcohol warnings focused on cancer risks in several European countries. We will use mixed methods (qualitative, quantitative and eye tracking) to examine how different formats and messages influence consumer attention, knowledge, awareness, risk perception, attitude and behavioural intentions across diverse cultural and consumer literacy contexts. As marketing can weaken the effect of alcohol warnings [25], the impact of warning labels will also be evaluated in real-world packaging and advertising contexts. Given the current lack of implementation of evidence-based European alcohol warnings in Europe or globally [26], we will analyse political and public discourse (e.g. media reports, parliamentary documents) to explore the influence of industry lobbying. Research from countries such as Canada, Ireland, Australia and France [27-29] has documented how alcohol industry lobbying has delayed or prevented implementation of effective labelling. We aim to conduct similar analysis of lobbying tactics in additional European countries.

The second component of objective (4) focuses on front-of-pack nutrition labelling. We will assess the impact of exposure to Nutri-Score [30] in online experimental supermarkets on purchasing behaviours across socioeconomically diverse groups. We will also analyse real-life purchase data from Belgium and France to assess levels of Nutri-Score exposure by socioeconomic status, dietary habits and shopping patterns. The analysis will explore associations between label exposure and overall dietary quality using panel and questionnaire data.

For objective (5), we will pilot new tools for monitoring digital marketing of unhealthy products, including high-fat, -sugar and -salt foods, alcoholic beverages, tobacco and breastmilk substitutes, as targeted at children, adolescents and mothers of young children. A screen-recording app/software will be tested in sub-samples of children and women with children under 36 months in three European countries. All recorded data will de-identified before analysis by quantitative and qualitative methods.

Finally, for objective (6), we will assess the impact of policy measures aimed at reducing air pollution, noise and exposure to environmental chemicals. This will involve mapping and evaluating existing policies and legislative frameworks and conducting burden-of-disease estimations in a cost–benefit analysis.

## Expected results

**Figure 1.**
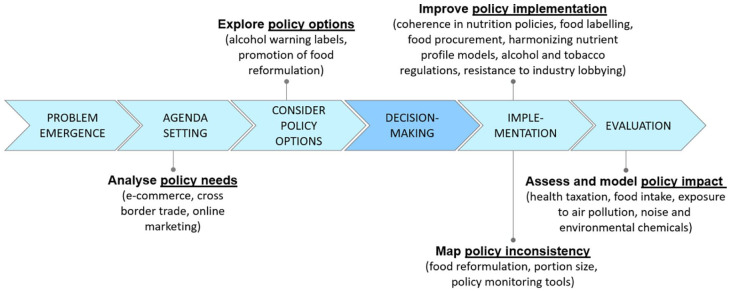
A schematic illustration of the link between the planned activities in the Joint Action PreventNCD (Joint Action on the Prevention of Cancer and Other Non-communicable Diseases) project and the stages of the public policy process.

We anticipate the results of this workstream will support policy-making processes in EU member states by strengthening and scaling up the use of regulatory measures to reduce the burden of NCDs (see Figure 1).

The anticipated outputs include *an assessment* of NCD and cancer prevention policies across EU member states, along with recommendations for emerging policy areas requiring enhanced monitoring, such as regulation of novel nicotine products. The project will *model the historical and projected impact of policies* on exposure to per- and polyfluoroalkyl substances in Europe.

Another key output will be a *report* with the evidence in the impacts of health taxation on tobacco, alcohol, food, non-alcoholic beverages and environmental pollutants. The project will contribute to the development of a *microsimulation model* that will enable EU member states to forecast consumption shifts from changes in taxation. Additionally, the project will also provide *recommendations* for regulation of e-commerce of tobacco and alcohol.

In collaboration with WHO, the project will propose a *harmonized nutrient profiling model* applicable to support a range of nutrition-related policies, including food marketing restrictions, food labelling, taxation and reformulation. It will also evaluate the potential of food reformulation strategies to reduce intake of sugars, saturated fats and salt. *Guidance* will be developed for sustainable PFP and standardized portion sizes, including sample weekly school menus for school-aged children.

The project will report on the scientific evidence on the effectiveness of *alcohol warning labelling*, including implementation *guidelines* and analysis of industry opposition. It will also generate new evidence of the impact *front-of-pack labelling* on consumer behaviour.

Furthermore, the project will establish *an EU-wide Implementation Package* to support member states to enact legislation to restrict harmful marketing practices, specifically targeting children and youth. This will be complemented by public awareness campaigns and the formation of stakeholder networks, including representatives from civil society and the education sector.

Finally, the project will support environmental regulation through a *report* modelling the effects of air pollution, noise and chemical pollution on NCD outcomes.

## Discussion

In this paper, we have outlined the objectives and content of the workstream within the JA PreventNCD project [17] that focus on regulatory and population-level interventions aimed at mitigating key risk factors, thereby reducing the burden of NCDs. The policy measures presented are aligned with the EU’s own strategic policy documents [18,19]. However, substantial disparities persist among European countries in terms of implementation, policy content and enforcement mechanisms.

These disparities can partly be attributed to the complexities of policy decision-making processes, which are influenced by vested interests, political agendas, ideological stances, public opinion and broader public discourse. Policy decisions must also balance potential benefits and harms, such as implications for health equity, alongside consideration of cost and cost-effectiveness, acceptability and feasibility [31]. Moreover, health outcomes are not always prioritized; issues such as security and economic stability currently dominate the political agenda. An overview of the regulative policy addressed in the project, along with their intended purposes, health equity implications and related policy domains, is provided in Table I.

**Table I.** Overview of regulative policy actions in the JA PreventNCD (Joint Action on the Prevention of Cancer and Other Non-communicable Diseases) project, including the purposes, health equity aspects, related policy areas and the foreseen contributions from the project.

Policy action	Purpose	Equity aspects	Potential co-benefit with other policy areas than health	JA PreventNCD contribution to policy decision making
Health taxation	Increase cost through excise taxes to reduce consumption of unhealthy commodities	The risk of adverse effects on low-income households will be accounted for [32]	Economy	Modelling the potential impact of different health taxation models will show the benefits
Regulation of e-commerce	Reduce e-commerce of unhealthy commodities, particularly of underaged and illegal products	The expected impact of regulation of e-commerce will be independent of socioeconomic status	Economy	Documentation of the extent and content of e-commerce of unhealthy commodities and needs for policy actions
Regulation of cross-border trade	Reduce cross-border trade to increase the impact of fiscal policies	Regulation of cross-border trade will impact population living close to borders, and travellers	Economy Foreign affairs	Documentation of the extent of cross-border trade and needs for policy actions
Recommendations for sustainable public food procurement	Ensure that public procurement of foods and beverages will be according to guidelines for healthy and sustainable foods	The expected impact of sustainable public food procurement on consumption will be independent of socioeconomic status [33]	Agriculture Environment Education	A new mechanism for organising public procurement of foods and beverages
Restriction of marketing	Protect children and youth from exposure to marketing of unhealthy commodities	The expected impact of marketing restrictions will be independent of socioeconomic status [32]	Education Children	Support the policy needs for regulation of exposure of unwanted marketing of unhealthy commodities to children and young people
A harmonized nutrient profile model	Provide a nutrient profile model to rank foods and beverages on the nutritional composition related to health	The model is made for the general population, and independent of socioeconomic status	Agriculture Education	Implementation of nutrient profile to support coherence in nutrition policies
Food reformulation	Reformulate and improve the nutrient content of existing foods and beverages	The expected impact of food reformulation will be independent of socioeconomic status [34]	Agriculture	Support reformulation of food and beverages as a policy option
Alcohol warning labelling	Increase the effectiveness of alcohol labels in a European cultural setting (with a focus on risks of cancer) Identify the lobbying of the alcohol industry to block the implementation of effective and mandatory warnings	The expected effect of alcohol warnings according to the level of health literacy [35]	Economy Education Environment Social welfare	Support the implementation of evidence-based alcohol warning labelling in Europe
Front-of-pack labelling (FoPL) of food products	Provide a mandatory, standardised and interpretative label that includes both positive and negative aspects to guide consumers	The risk that FoPL widening social inequalities will be accounted for [32]	Agriculture	Support implementation of FoPL in Europe
Policies to reduce air pollution, noise and environmental chemicals	Reduce risks for NCDs and cancers caused by air pollution, noise and environmental chemicals	Policies will be assessed with an equity lens	EconomyEducationEnvironmentHousing	Modelling the potential impact on health of policies targeting air pollution, noise and environmental chemicals
Policy action monitoring tools	Providing an overview of policy actions related to prevention of NCDs, and cancers will demonstrate policy differences between member states	Policies will be assessed with an equity lens	AgricultureEconomyEducationEnvironment	Documentation of policy differences and incoherences between European Union members and lack of policies

JA PreventNCD, Joint Action Prevent Non-communicable Diseases; NCD, non-communicable diseases.

A key strength of the workstream on regulatory and fiscal measures is that the policy options promoted are already referenced in EU-level policy documents. Within the workstream, greater emphasis is placed on certain risk factors, such as dietary behaviour, partly due to the legacy of earlier initiatives like the JA BestRemap [23] project which focused specifically on diet. This emphasis also reflects the interests and priorities expressed by participating countries and institutions participating in the JA PreventNCD project.

Many planned activities in the workstream address multiple risk factors simultaneously, for example, those concerning health taxation and the regulation of digital marketing. In addition to generating new evidence and documentation to support policy decision making, it is important to acknowledge that the impact of such processes often extends beyond the 4-year timeframe of the project (2024–2027). A particular advantage of addressing regulatory measures through the Joint Action mechanism is the facilitation of collaborative efforts across European countries. Challenges such as air pollution and environmental contaminants transcend national borders, making collaborative action imperative. Similarly, challenges such as curbing cross-border trade in unhealthy commodities and enhancing the viability and effectiveness of alcohol warnings cannot be effectively addressed at the national level alone. In this context, documenting and comparing national policy differences through systematic policy monitoring and mapping is especially valuable for promoting accountability and encourage evidence-based policy-making alignment.

Despite its strengths, the workstream may encounter several key challenges. Foremost among these is the reliance on political will, which may vary widely across member states. It is also essential that all proposed policy measures undergo a thorough equity assessment to ensure they do not widen existing social inequalities but instead contribute to their reduction. Another challenge lies in the reliability and accuracy of data collection. The success of mapping and survey activities will depend on access to relevant, high-quality data and the identification of appropriate stakeholders and national experts.

While the workstream is expected to generate substantial evidence to inform policy development, its impact will rely on sustained engagement with policymakers, interest groups, civil society and citizens. The relevance and potential impact of the project are further reinforced by its foundation in a collaboration involving over 100 national authorities from the 25 participating countries [17]. Moreover, the project includes a dedicated workstream on ensuring the long-term sustainability of its outputs and their integration into national and EU-level policy processes [31].

## Conclusion

Regulatory policy measures for the prevention of NCDs are well established and widely recommended, yet many European countries have not implemented them, despite their inclusion in EU policy frameworks and endorsement by institutions, including WHO. The JA PreventNCD project aims to strengthen compliance, coherence, implementation and enforcement of these measures by generating and disseminating actionable evidence. Through this effort, the project seeks to support member states in making informed policy decision processes to reduce the preventable burden of NCDs across Europe.
